# Prenatal mercury exposure and features of autism: a prospective population study

**DOI:** 10.1186/s13229-018-0215-7

**Published:** 2018-04-23

**Authors:** Jean Golding, Dheeraj Rai, Steven Gregory, Genette Ellis, Alan Emond, Yasmin Iles-Caven, Joseph Hibbeln, Caroline Taylor

**Affiliations:** 1 Centre for Child and Adolescent Health, Bristol Medical School, University of Bristol, Oakfield House, Oakfield Grove, Bristol, BS8 2BN UK; 2 Centre for Academic Mental Health, Bristol Medical School, University of Bristol, Oakfield House, Oakfield Grove, Bristol, BS8 2BN UK; 3 Section on Nutritional Neurosciences, LMBB, National Institute on Alcohol Abuse and Alcoholism, National Institutes of Health, 31 Center Drive 1B/58, Bethesda, MD 20892 USA

**Keywords:** ALSPAC, Prenatal mercury, Fish consumption, Autism, Autistic traits, Social cognition, Dental amalgam

## Abstract

**Background:**

Mercury (Hg) has been suspected of causing autism in the past, especially a suspected link with vaccinations containing thiomersal, but a review of the literature shows that has been largely repudiated. Of more significant burden is the total quantity of Hg in the environment. Here, we have used the Avon Longitudinal Study of Parents and Children (ALSPAC) to test whether prenatal exposure from total maternal blood Hg in the first half of pregnancy is associated with the risk of autism or of extreme levels of autistic traits. This is the largest longitudinal study to date to have tested this hypothesis and the only one to have considered early pregnancy.

**Methods:**

We have used three strategies: (1) direct comparison of 45 pregnancies resulting in children with diagnosed autism from a population of 3840, (2) comparison of high scores on each of the four autistic traits within the population at risk (*n*~2800), and (3) indirect measures of association of these outcomes with proxies for increased Hg levels such as frequency of fish consumption and exposure to dental amalgam (*n* > 8000). Logistic regression adjusted for social conditions including maternal age, housing circumstances, maternal education, and parity. Interactions were tested between risks to offspring of fish and non-fish eaters.

**Results:**

There was no suggestion of an adverse effect of total prenatal blood Hg levels on diagnosed autism (AOR 0.89; 95% CI 0.65, 1.22) per SD of Hg (*P* = 0.485). The only indication of adverse effects concerned a measure of poor social cognition when the mother ate no fish, where the AOR was 1.63 [95% CI 1.02, 2.62] per SD of Hg (*P* = 0.041), significantly different from the association among the offspring of fish-eaters (AOR = 0.74 [95% CI 0.41, 1.35]).

**Conclusion:**

In conclusion, our study identifies no adverse effect of prenatal total blood Hg on autism or autistic traits provided the mother ate fish. Although these results should be confirmed in other populations, accumulating evidence substantiates the recommendation to eat fish during pregnancy.

**Electronic supplementary material:**

The online version of this article (10.1186/s13229-018-0215-7) contains supplementary material, which is available to authorized users.

## Background

The possible link between Hg exposure and autism has attracted much controversy and debate over many years, largely related to suggestions that the Hg containing the additive thiomersal (thimerosal) in immunizations was causing harm (e.g., [1]). Reviews of the literature of accumulated evidence have since indicated a lack of association [2–4], and these have gradually reduced the general fear of having the baby immunized. In actual fact, the amount of Hg in thiomersal was relatively low compared with the amount absorbed from the atmosphere, the diet, and dental amalgam [5]. Nevertheless, there is still a fear concerning exposure to mercury among pregnant women, particularly focused around the consumption of seafood [6].

Although there are undoubtedly severe adverse effects with exposure to very high levels of mercury, prospective studies (summarized in the “Discussion” section of this paper) have mostly shown no adverse effects at a population level. Nevertheless, there is still confusion worldwide between the possible adverse effects on the offspring of low levels of mercury in pregnancy, especially when the exposure is from seafood. We have carried out a series of studies that have compared the levels of total mercury in maternal prenatal blood and shown that among children born to mothers who ate fish, there were no adverse associations with outcomes such as child development, child behavior, high blood pressure, or suboptimal IQ level [7–10].

## Methods

### The aim

Since there have been relatively few studies determining whether there is any association between total prenatal Hg exposure during pregnancy and autism, we have used a large population-based study in England, to determine whether (1) maternal prenatal whole blood Hg levels or (2) indirect measures of fetal exposure to Hg were associated with either a diagnosis of autism or the component traits of autism.

### The population

Avon Longitudinal Study of Parents and Children (ALSPAC) is a pre-birth cohort study that enrolled ~ 80% of pregnant women resident in the Avon area of the UK in 1991–1992. The aim of the study was to assess ways in which the environment (defined in its broadest sense) interacted with genetics to influence the health, development, and well-being of the offspring. To this end, data collection used a variety of methodologies including direct examination of the offspring; self-completion questionnaires administered to the parents, the children, and their teachers; collection and assays of biological samples (including DNA); and linkage to health and education records [11, 12]. The study website contains details of all the data that are available through a fully searchable data dictionary: [http://www.bris.ac.uk/alspac/researchers/data-access/data-dictionary/].

### The exposures

#### Prenatal measures of total mercury

Blood samples deliberately collected in acid-washed containers for determination of trace metals were obtained from 4484 women residing in two of the three Health Authority areas of the recruitment region. Samples were collected by midwives as early as possible in pregnancy. The sociodemographic characteristics of the women who donated samples were comparable to those of the rest of the ALSPAC study population apart from including a slight excess of older and more educated mothers [13]. Gestational age at sample collection [known for 4472 mothers (99.7%)] had a median value of 11 weeks and mode of 10 weeks. The interquartile range (IQR) was 9–13 weeks, and 93% of the samples were collected at < 18 weeks gestation. Samples were stored for 0–4 days at 4 °C at the collection site before being sent to the central Bristol laboratory. Samples were transported at room temperature for up to 3 h and stored at 4 °C as whole blood in the original collection tubes for 18–19.5 years before analysis [14].

Analysis of the blood samples for whole blood Hg were carried out by the laboratory of Dr. Robert Jones at the Centers for Disease Control and Prevention (CDC) [CDC method 3009.1; unpublished information]. Clotted whole blood was digested to remove all clots, before being analyzed using the inductively coupled plasma dynamic reaction cell mass spectrometry (ICP-DRC-MS) [15, 16]. The entire amount of clotted whole blood was transferred to a digestion tube using concentrated nitric acid with the volume estimated from the weight. The blood sample was heated in a microwave oven at a controlled temperature and time, during which the organic matrix of the blood was digested removing the clots. ICP-DRC-MS internal standards (iridium and tellurium) were added at a constant concentration to all blanks, calibrators, and samples (at the time of 1:9 dilution of digestate) to facilitate correction for instrument noise and drift. The standard additions method of calibration was used to optimize the analytical sensitivity of the method for the whole blood samples. A recovery spike was included in each analytical run for calibration verification and as a blind quality control (QC) sample. The ICP-DRC-MS was operated in the DRC mode using oxygen when analyzing for Hg. QC materials as well as in-house QC samples with control limits unknown to the analysts were used for daily quality control. The level of detection (LOD) for Hg was 0.24 μg/L; three samples were below this level and were ascribed a value of 0.7 times the LOD (since the frequency distribution of Hg had evidence of a lower tail, a factor greater than 0.5 was deemed appropriate to reflect the likelihood that more of these three results would be closer to the LOD than zero). The maternal blood Hg levels ranged from 0.17 to 12.76 μg/L, with 5th, 10th, 50th, 90th, and 95th centiles of 0.81, 0.99, 1.86, 3.33, and 4.02 μg/L, respectively.

#### Proxy exposure measures

We have shown elsewhere that the blood Hg level increased if the woman ate fish during pregnancy [14] and with the number of amalgam fillings in the mouth and whether she had dental treatment involving amalgam during pregnancy [17] (see Additional file 1). We have therefore analyzed these variables as proxies for maternal Hg level.

The measures of fish consumption were obtained during pregnancy and comprised three questions concerning the frequency with which the mother ate (a) white fish, (b) oily fish, and (c) shellfish. Options given were as follows: not at all, about once in 2 weeks, 1–3 times a week, 4–7 times a week, and more than once a day. Dental exposures were also obtained from questionnaires completed by the woman in her own home and posted back to the study. They comprised questions concerning (i) whether she had an amalgam filling inserted during pregnancy, (ii) whether she had an amalgam filling extracted during pregnancy, and (iii) approximately how many amalgam fillings were in the woman’s mouth at the time she was pregnant.

### Outcome measures

#### Autistic traits

We have used the four independent trait predictors of autism identified previously as most predictive of autism in this cohort and described in the Appendix. They include measures of social communication at age 7 (using the Social and Communication Disorders Checklist (SCDC)), coherent speech (using the Child Communication Checklist) at age 9, sociability (using the Emotionality, Activity, Sociability temperament traits (EAS) temperament scale) at 3 years, and a derived repetitive behavior measure at 5. Each has been shown to be an independent predictor of clinically identified autism in ALSPAC using health records [18, 19]. Since these continuous scales were highly skewed and not easily amenable to transformation, we dichotomized them in order to identify children with approximately the worst 10% scores as described elsewhere [20] (details also provided in the Appendix). These extreme 10% subgroups of the traits are referred to as having poor social cognition, poor coherence, poor sociability, and repetitive behavior.

#### Identification of autism

In order to identify the children with autism, we used the following sources: (a) a review of all children given a statement for special educational provision in the Avon area to identify children diagnosed as on the autism spectrum using the ICD-10 criteria [18]; (b) the mother’s answer to the question at age 9 “Have you ever been told that your child has autism, Asperger’s syndrome or autistic spectrum disorder”; (c) classification as Pervasive Development Disorder using questions from the DAWBA questionnaire at 91 months [21], with the answers to the questionnaire classified by a child psychiatrist; (d) text responses to any question on diagnoses given to the child in questionnaires from 6 months to 11 years; and (e) letters from parents to the Study Director with details of the child’s diagnosis. We used all sources. We have previously cross-validated ASD cases confirmed only by maternal report by showing that they have strong associations with various autistic trait measures [20]. This method identified 177 offspring (139 boys, 38 girls) with a presumed diagnosis of autism by age 11, giving a prevalence of 1.3%.

### Confounders used in the adjusted analyses

The following factors collected using self-completion questionnaires during pregnancy were used as potential confounders in the analyses of autistic traits: maternal age, parity (number of previous pregnancies resulting in a live or stillbirth), family adversity index, housing tenure, household crowding, life events, smoking in pregnancy, and prenatal alcohol consumption. In addition, we took account of whether the child was breastfed, as in our previous studies [7, 10]. Although there were too few children with diagnosed autism to allow for a large number of confounders, we have allowed for the key variables collected prenatally that have influenced whether a diagnosis has been given; these comprise mother’s age, education level, housing tenure, time lived in Avon, and maternal locus of control.

### Statistical analyses

There were two sets of analyses:

Analyses A comprised the assessment of maternal prenatal total blood mercury in regard to autistic outcomes.

Analyses B examined the associations between proxies for mercury exposure (seafood consumption and dental amalgam) and the autistic outcomes.

The five outcomes in both sets of analyses comprised the binary measures involving the most extreme 10% of the autistic traits as well as the children with diagnosed autism. Logistic regression was used to assess the association between each of the five outcomes and (A) direct maternal total blood mercury levels in pregnancy and (B) the proxy measures indicating increased levels of blood mercury.

For adjustment using analyses A for the autistic traits, the models first included the confounders outlined above and then added the measure of mercury. In addition, since we have shown interactions between prenatal fish consumption and total blood Hg in predicting the offspring IQ [7], we stratified by maternal fish consumption and examined the results for interactions between Hg levels and whether the mother consumed fish, for each autistic trait. Further analyses using proxies for Hg exposure in pregnancy (seafood intake and dental amalgam experience) adjusted for the same set of possible confounders but did not look for interactions (see Table 4).

## Results

### Biases between the population for whom blood mercury was available and the rest of the cohort

We have shown elsewhere that there were no differences between the women for whom a trace metal result was obtained and the rest of the population in relation to their seafood intake [14], dental treatment [17], social conditions, and lifestyle [9], with two exceptions: more educated and older women were more likely to have had blood taken for trace metal analyses.

### Variation of diagnosed autism with prenatal whole blood mercury

Of the 177 pregnancies that resulted in a child diagnosed with autism, 45 had a measure of total blood Hg. The mean blood level of Hg in this group [2.15 (SD 0.95) μg/L] was similar to the level in the remaining 3840 pregnancies [2.08 (SD 1.09) μg/L, *P* = 0.655].

Table 1 demonstrates the distribution of the pregnancies within quintiles of maternal prenatal total blood Hg (with the upper quintile divided into the two upper deciles) by autism outcome. There was no evidence for a trend of increasing prevalence with increasing level of blood Hg, and the highest decile of the distribution (> 3.39 μg/L) had one of the lowest prevalences of autism (1.09% of children of women with the highest levels compared with 1.34% of the children of women with the lowest blood mercury levels were diagnosed with autism). The adjusted regression analysis for autism using the five possible confounders (mother’s age, education level, housing tenure, time lived in Avon, and maternal locus of control) found no evidence of an association between increased Hg levels and an autism diagnosis [adjusted OR 0.89; 95% CI 0.65, 1.22 per SD increase in Hg (*P* = 0.485)].

**Table 1:** Proportion of children to have diagnosed autism or extreme levels on autistic trait measures within each group of increasing prenatal total mercury levels

Prenatal blood mercury (μg/L)^a^	Diagnosed autism, % (*n*)	Autistic traits, % (*n*)			
		Poor social cognition	Poor sociability	Poor coherence	Repetitive behavior
≤ 1.28	1.34 (11)	12.7 (51)	12.7 (68)	12.4 (48)	6.6 (30)
1.29–1.68	0.51 (4)	12.2 (53)	12.6 (70)	10.7 (45)	6.9 (32)
1.69–2.10	0.67 (5)	11.8 (56)	11.3 (66)	7.8 (35)	4.7 (24)
2.11–2.74	1.81 (14)	11.3 (58)	11.1 (68)	9.4 (46)	5.9 (33)
2.75–3.39	1.79 (7)	9.6 (24)	10.4 (32)	12.3 (31)	7.4 (20)
> 3.39	1.09 (4)	13.0 (34)	10.5 (32)	8.4 (21)	2.6 (7)
All affected^b^	1.16 (45)	11.8 (276)	11.6 (336)	10.0 (226)	5.8 (146)
Total *N*	3885	2333	2902	2249	2529
*P* (5df)	0.112	0.840	0.827	0.181	0.102

*df* degrees of freedom

^a^First four quintiles and the last two deciles

^b^Note that overall the percentage of extreme autistic traits varies—it is as near to 10% as possible

### Associations of autism traits with prenatal blood mercury

The correlation coefficients [95% confidence interval] between increasing maternal mercury level and increasing level of autistic trait were as follows: social cognition *r* = − 0.02 [− 0.06, + 0.02]; sociability *r* = − 0.04 [− 0.08, − 0.01]; coherence *r* = − 0.03[− 0.07, + 0.01]; and repetitive behavior *r* = − 0.04 [− 0.08, + 0.001]. Thus, all correlations indicated that with increasing levels of mercury, the signs of autism were slightly less, but none were statistically significant. It was also apparent from Table 1 that there was no evidence of an increasing prevalence of any of the extreme levels of autistic traits with increasing prenatal blood Hg. Table 2 shows the unadjusted and adjusted odds of total Hg levels with the dichotomized autism trait measures; the associations significant at the 10% level are italicized. For poor sociability, there were many significant unadjusted associations with Hg level, but only one survived adjustment—and that was only of borderline significance (*P* = 0.073); all the results indicated that high levels of prenatal Hg were associated with reduced risk of poor sociability. Neither poor coherence nor repetitive behavior was associated with prenatal Hg at the 0.10 level of significance. The only adjusted association of statistical significance at the 0.05 level concerned the relationship between Hg and poor social cognition among the offspring of women who did not eat fish; this relationship was significantly different from the women who did eat fish.

**Table 2:** Unadjusted and adjusted odds ratios (OR [95%CI] per SD of mercury) between prenatal total blood mercury and the extreme levels of autistic traits are shown together with the results of separate analyses for children of mothers who did and those who did not eat fish during pregnancy

Population	Autistic trait			
	Poor social cognition	Poor sociability	Poor coherence	Repetitive behavior
All offspring				
Unadjusted	0.96 [0.87, 1.06]	*0.83 [0.74, 0.93]*	0.96 [0.86, 1.07]	0.94 [0.87, 1.02]
*N* (*P*)	2331 (0.432)	*2898 (0.002)*	2249 (0.411)	2528 (0.167)
Adjusted	0.96 [0.85, 1.08]	*0.88 [0.77, 1.01]*	1.01 [0.89, 1.14]	0.94 [0.86, 1.04]
*N* (*P*)	1991 (0.459)	*2422 (0.073)*	1938 (0.912)	2162 (0.234)
Mother ate fish				
Unadjusted	0.95[0.84, 1.06]	*0.85 [0.75, 0.97]*	1.00 [0.89, 1.13]	0.95 [0.86, 1.04]
*N* (*P*)	1945 (0.355)	*2389 (0.015)*	1873 (0.985)	2095 (0.228)
Adjusted	0.92[0.80, 1.05]^a^	0.89 [0.76, 1.03]	1.04 [0.91, 1.19]	0.93 [0.84, 1.03]
*N* (*P*)	1744 (0.220)	2104 (0.106)	1698 (0.560)	1884 (0.150)
Mother ate no fish				
Unadjusted	1.26 [0.84, 1.87]	*0.64 [0.39, 1.05]*	0.96 [0.64, 1.45]	1.22[0.90, 1.64]
*N* (*P*)	285 (0.261)	*373 (0.079)*	273 (0.850)	317 (0.197)
Adjusted	*1.63 [1.02, 2.62]* ^b^	0.74 [0.41, 1.35]	0.87 [0.51, 1.48]	1.16 [0.81, 1.66]
*N* (*P*)	*240 (0.041)*	280 (0.327)	231 (0.598)	272 (0.425)

All associations with *P* <  0.10 are italicized

^a^Adjusted for maternal age, parity, family adversity index, housing tenure, household crowding, life events, smoking in pregnancy, prenatal alcohol consumption, and whether child was breastfed

^b^Significant interaction between adjusted results for offspring of fish and non-fish eaters

### Associations with proxies of mercury level

Table 3 shows the prevalence of diagnosed autism and the extreme levels of autism traits according to the frequency with which the pregnant women ate white fish, oily fish, and shellfish. No differences were apparent for diagnosed autism or the repetitive behavior trait, but children with mothers reporting eating no white fish appeared to have the highest prevalence of impairments in social cognition (16.1% vs 12.5 and 11.9%; *P* < 0.001) and coherence (12.1% vs 9.4 and 9.7%; *P* = 0.026). There were mixed findings for poor sociability.

**Table 3:** Proportion of children with diagnosed autism or extreme levels of autistic traits by proxies for increased mercury exposure

Proxy for mercury exposure	Diagnosed autism, % (*n*)	Autistic traits, % (*n*)			
		Poor social cognition	Poor sociability	Poor coherence	Repetitive behavior
White fish frequency^a^					
Not at all	1.21 (27)	*16.1 (204)*	*11.6 (191)*	*12.1 (147)*	6.5 (90)
Once in 2 weeks	1.33 (65)	*12.5 (386)*	*10.4 (397)*	*9.4 (277)*	6.0 (199)
> once a week	1.37 (69)	*11.9 (397)*	*12.3 (494)*	*9.7 (314)*	5.6 (197)
*P* (2df)	0.640	*< 0.001*	*0.029*	*0.026*	0.481
Oily fish frequency^a^					
Not at all	1.23 (63)	*13.8 (407)*	*12.6 (480)*	*10.9 (308)*	6.0 (195)
Once in 2 weeks	1.49 (60)	*13.2 (354)*	*10.3 (334)*	*10.0 (256)*	5.8 (165)
> once a week	1.27 (38)	*11.1 (226)*	*11.0 (268)*	*8.7 (174)*	5.9 (126)
*P* (2df)	0.672	*0.017*	*0.010*	*0.044*	0.971
Shellfish frequency^a^					
Not at all	1.35 (132)	13.0 (801)	*11.0 (834)*	10.2 (598)	5.8 (384)
Any	1.23 (29)	12.1 (186)	*13.1 (248)*	9.4 (140)	6.3 (102)
*P* (2df)	0.527	0.352	*0.008*	0.414	0.438
Had amalgam fillings inserted in pregnancy					
Yes	1.09 (12)	12.8 (222)	*10.0 (213)*	10.0 (169)	*6.7 (127)*
No	1.52 (67)	12.8 (700)	*11.7 (786)*	9.9 (516)	*5.7 (332)*
*P* (1df)	0.283	0.995	*0.029*	0.920	*0.092*
Had amalgam fillings removed in pregnancy					
Yes	1.03 (15)	13.8 (155)	*9.1 (126)*	10.6 (118)	6.0 (73)
No	1.58 (126)	12.6 (767)	*11.7 (873)*	9.8 (567)	5.9 (386)
*P* (1df)	0.110	0.284	*0.006*	0.405	0.865
Number of amalgams in mouth in pregnancy					
0	1.62 (11)	13.5 (60)	*12.4 (77)*	11.4 (47)	6.7 (34)
1–3	1.29 (25)	13.5 (188)	*14.0 (250)*	8.6 (112)	6.8 (101)
4+	1.57 (96)	12.6 (612)	*10.4 (600)*	9.9 (463)	5.9 (305)
*P* (2df)	0.677	0.653	*< 0.001*	0.196	0.355

All associations with *P* < 0.10 are italicized

*df* degrees of freedom

^a^Amount consumed by mother as reported at 32 weeks gestation

In regard to dental features, poor sociability appeared to show significant relationships, but all were such that increased maternal exposure to dental amalgam was associated with lower rates of extreme levels of autistic traits. Upon adjustment (Table 4), there were four significant associations at the 10% level; all indicated a protective effect associated with the exposures that would have increased the mothers’ Hg level (see Additional file 1).

**Table 4:** Adjusted associations between prenatal mercury and offspring diagnosed autism and extreme levels of autistic traits. Odds ratios [95%CI] adjusted for maternal age, family adversity index, prenatal life events, smoking and alcohol in pregnancy, maternal locus of control, and maternal education

Proxy for Hg exposure^a^	Diagnosed autism, OR [95% CI]	Autistic trait, OR [95% CI]			
		Poor social cognition	Poor sociability	Poor coherence	Repetitive behavior
White fish	1.39 [0.80, 2.40]	*0.85 [0.71 ,1.03]*	0.93 [0.77, 1.11]	0.90 [0.78, 1.04]	1.01 [0.88, 1.16]
	(*P* = 0.238)	*(P = 0.092)*	(*P* = 0.399)	(*P* = 0.163)	(*P* = 0.886)
Oily fish	1.00 [0.68, 1.47]	0.98 [0.84, 1.14]	0.90 [0.78, 1.04]	1.01 [0.90, 1.14]	1.03 [0.92, 1.15]
	(*P* = 0.994)	(*P* = 0.774)	(*P* = 0.150)	(*P* = 0.853)	(*P* = 0.613)
Shellfish	0.86 [0.54, 1.38]	0.92 [0.77, 1.11]	0.91 [0.76, 1.08]	0.94 [0.81, 1.08]	1.03 [0.90, 1.17]
	(*P* = 0.542)	(*P* = 0.401)	(*P* = 0.284)	(*P* = 0.351)	(*P* = 0.707)
Amalgam inserted	*0.62 [0.37, 1.03]*	0.99 [0.83, 1.18]	1.14 [0.97, 1.34]	1.07 [0.94, 1.22]	1.02 [0.90, 1.16]
	*(P = 0.067)*	(*P* = 0.905)	(*P* = 0.119)	(*P* = 0.320)	(*P* = 0.761)
Amalgam extracted	*0.47 [0.24, 0.93]*	1.09 [0.89, 1.33]	1.14 [0.94, 1.38]	1.10 [0.94, 1.28]	1.04 [0.89, 1.20]
	*(P = 0.031)*	(*P* = 0.424)	(*P* = 0.178)	(*P* = 0.237)	(*P* = 0.647)
No. of amalgam fillings	0.96 [0.76, 1.21]	1.00 [0.91, 1.09]	*0.91 [0.84, 0.99]*	1.01 [0.94, 1.09]	0.95 [0.89, 1.01]
	(*P* = 0.730)	(*P* = 0.965)	*(P = 0.033)*	(*P* = 0.790)	(*P* = 0.122)
No. in analyses	7498–10,452	5888–6972	7119–8512	5646–6690	6302–7477

All associations with *P* < 0.10 are italicized

^a^Categorization of variables as shown in Table 3. Significance levels calculated testing for a linear trend

## Discussion

In this large birth cohort study with prospectively collected information, we found no evidence to suggest that prenatal exposure to total maternal blood Hg, measured directly in whole blood, and indirectly through fish consumption and dental amalgam fillings, was associated with autism or increased autism symptoms in the offspring.

There is increasing recognition that trying to find a biological basis for syndromes such as autism is probably best served by studying the component traits [22, 23], on the assumption that particular component traits may be influenced by different environmental and/or genetic factors. Here, we have shown a differential relationship between the social cognition trait and prenatal Hg exposure, such that there was a significant difference in apparently protective effects contingent upon whether the mother ate fish. This was not found for the other traits and may imply that this trait is particularly influenced by the beneficial components of fish such as the omega-3 fatty acids, iodine, and vitamins D and B2.

### Comparison with the literature

There have been several reviews showing no adverse associations between autism and ethyl Hg in thiomersal, but they have pointed out that the studies looking at other Hg exposures tend to have concentrated on either air pollutants or dental or dietary exposures but rarely looked at Hg biomarkers [24, 25] apart from one study of 84 cases of autism and 158 controls which showed no difference in mid-pregnancy serum or cord blood Hg levels [26]. Our study is consistent with these prior null findings, with the additional advantage of being able to assess the effect of direct as well as indirect measures of Hg exposure on the diagnosis as well as on four different autistic traits.

Although it did not consider diagnosed autism, a study that bears the closest resemblance to our own analyzed data comprising a longitudinal birth cohort in the Seychelles where the consumption of ocean fish is almost universal and the prenatal Hg levels are about 10 times those of the USA. Hair collected from 537 mothers shortly after birth was assayed for Hg, and levels were assumed to be a proxy for prenatal Hg exposure of the fetus. The study found no evidence of a deleterious effect of these Hg levels or of fish consumption with measures of social interaction or communication in the offspring [27].

Our findings of an interaction with prenatal fish consumption are mirrored by a study of 2062 children tested for IQ using the WISC at 8 years of age [9]; after detailed adjustment, there was a difference of 3 IQ points per SD of Hg between children of fish eaters (+ 0.83) and non-fish eaters (− 2.22) (*P* = 0.043). This difference, and that with social cognition found here, suggests that the benefits of nutrients in fish counteract any possible adverse cognitive and behavioral differences that may be caused by prenatal exposure to Hg.

### Strengths and limitations

There are a number of limitations of this study: (i) Despite the large sample, the numbers of autism cases with prenatal total blood Hg measured were relatively low, limiting statistical power. (ii) Although we accounted for several important confounders which are relevant to Hg levels and autism, the possibility of unmeasured confounding cannot be ruled out. (iii) The measures of Hg were obtained from whole blood in the first half of pregnancy—while having measures of Hg in early pregnancy may be a strength considering many teratogens are known to affect development at this stage, it may also be possible that exposure at later time points is more deleterious in regard to the autism spectrum. (iv) The data collected on fish consumption distinguished between oily and white fish but did not further characterize the types of fish consumed. Thus, we cannot identify the mothers who consumed fish at the extreme end of the food chain such as shark. However, although the levels of mercury in these fish are considerably greater than that in less predatory fish, there is no evidence of harmful effects to the fetus from eating such fish, as evidenced by the findings in this study of a lack of increasing risk to autism or autistic traits with increasing levels of maternal mercury. (v) The levels of total blood Hg in this population may be different from other populations, and therefore, caution is required before generalizing the results. For example, the median total blood Hg level in ALSPAC was 1.86 μg/L compared with 0.89 μg/L in the National Health and Nutrition Examination Survey (NHANES) in 1999–2000, but the proportion of women with higher than the recommended USA action level (5.8 μg/L) [28] was 8% in NHANES compared with only 1% in ALSPAC [14]. (vi) The blood used for analysis had been kept in the vacutainers in which they were collected for 19 years before assay. It is conceivable that some of the mercury might have leaked through the rubber stoppers. However, this is unlikely to have been differential in relation to the outcomes being studied and therefore could theoretically bias the relationship between maternal mercury and offspring outcome towards the null. (vii) The identification of cases of autism was not carried out using a specific examination but rather used a multisource ascertainment approach; consequently, the possibility of outcome misclassification cannot be ruled out. Nevertheless, we have previously validated additional cases identified against autistic symptoms [20] and have also found that polygenic risk scores for ASD from the most recent genome-wide association study with publicly available summary data [29] are associated with the ASD diagnosis identified in ALSPAC (paper under review).

On the other hand, this study provides a number of advantages: (a) it is based on a geographic population with a high enrolment rate (~ 80%) and consequently may be more generalizable to areas with similar distributions of blood Hg among pregnant women; (b) a relatively large number of confounders were available to be taken into account, thus diminishing the likelihood of bias in the results; (c) the prenatal data were collected prospectively with no knowledge as to how the child would develop, again reducing the likelihood of possible bias; and (d) sufficient numbers were available to allow comparison between offspring of fish and non-fish consumers for autistic traits (although not for diagnosed autism).

## Conclusions

In conclusion, this study did not find evidence to suggest that total prenatal blood Hg levels, or proxies for Hg levels, were important in relation to offspring diagnosis of autism. Although the results for the social communication trait mirrored results we have found for suboptimal IQ in showing an adverse effect of blood mercury if the mother ate no fish, but a beneficial association when fish was eaten, it is important that this be tested in other populations. There is no consistent evidence from this study to implicate prenatal exposure to mercury in the etiology of autism.

This is the largest prospective population study to date to address this question. It is the only study to compare total blood mercury levels in the first half of pregnancy among offspring with autism or high scores on autistic traits. It is also the only study to determine whether the exposures known to result in increased mercury levels were associated with autistic outcomes in the offspring.

### Additional file

Additional file 1: **Table S1**. Levels of maternal blood mercury above the 80th centile found with the proxies for mercury exposure as used in Table 4. (DOCX 16 kb)

## Appendix

### Independent trait descriptors of autism used in ALSPAC

#### The social communication trait

We used the 12-item Social and Communication Disorders Checklist (SCDC), developed by [30]. They showed that the internal consistency was excellent (0.93) and the test-retest reliability was high (0.81). The method was developed on clinical samples, and when later used on the ALSPAC population at age 7.7 years, the high end of the scale was shown to predict a variety of adverse outcomes but was most specific for autism spectrum disorder [31]. Further research with ALSPAC data showed that the measure was reasonably stable over time [32].

For the present analysis, we have used the prorated score, which was calculated when any items were missing a response by using the average of the items that had been answered by the individual (2.7% of the population, almost all of whom had just one item missing). If all items were missing, the score was put to missing. The measure ranged from 0 to 24; the higher the score the more impaired was the child’s social cognition. The distribution was skewed with a long upper tail. (12.8% had a score of over 6 and comprise the abnormal group for these analyses.)

#### The coherence measure

At age 9, the study mother completed a questionnaire which included seven of the nine scales of the first version of the Children’s Communication Checklist (CCC) [33]. This checklist was designed to assess aspects of communication that are not readily assessed by conventional standardized tests including aspects of speech and syntax, as well as pragmatic aspects such as over-literal interpretation of stereotyped language. Although the CCC was initially designed to identify pragmatic difficulties, it has been shown to be good at discriminating a wide range of language and communication problems from typical development [34]. Analyses of traits predictive of autism in ALSPAC showed that the Coherence scale performed better than the other scales [19] and consequently it is used here. The scale comprises eight items (e.g., “It is sometimes hard to make sense of what she is saying because it seems illogical or disconnected” and “She has difficulty in telling a story, or describing what she has done in a sequence of events”). The score ranged from 20 to 36, with higher scores indicating more typical behavior. The score had a skewed distribution. The lower tail used in this analysis comprised those children scoring ≤ 33 points (10.0% of the population).

#### Abnormal and repetitive behavior

This scale was developed from the answer to four questions in the questionnaire sent to the mother at 69 months; these were as follows: “How often does he/she (a) repeatedly rock his head or body for no reason; (b) have a tic or twitch; (c) have other unusual behavior”; or (d) “Does he/she stumble or get stuck on words, or repeat them many times? (e.g., I I I I want a sweet)”? The responses to each question were coded as often/always = 3; sometimes = 2; never = 1 and summed. The resultant scale had a range from 4 to 12, with 22% scoring 5 and only 5.9% scoring more than 5. Thus, it was impossible to approximate to a 10% cut-off; we therefore used > 5 as our abnormal group.

#### Sociability temperament

The questionnaire concerning the child sent to the study mothers when the child was 38 months of age included the 20 questions of the EAS temperament scale [35] and measured four traits—emotionality, activity, shyness and sociability—each based on the answers to 5 questions. The range of the Sociability sub-score was from 5 to 25 and the frequency distribution was approximately normal, a high score indicating a high level of sociability. The prorated scale was calculated for missing values as in the scales mentioned above. We then selected the lowest 11.4% of the children for our analyses (score < 8) as being the nearest to 10%.

## Abbreviations

ALSPAC: Avon Longitudinal Study of Parents and Children
AOC: Adjusted odds ratio
CDC: Centers for Disease Control and Prevention
CI: Confidence interval
DAWBA: Development and well-being assessment
DNA: Deoxyribonucleic acid
EAS: Emotionality, Activity, Sociability temperament traits
ICD-10: International Classification of Diseases, 10th Edition
ICP-DRC-MS: Inductively coupled plasma dynamic reaction cell mass spectrometry
IQ: Intelligence quotient
IQR: Interquartile range
LOD: Level of detection
NHANES: National Health and Nutrition Examination Survey
OR: Odds ratio
QC: Quality control
SD: Standard deviation
