# Supplementary material for: Prenatal mercury exposure and features of autism: a prospective population study
Source: Mol Autism. 2018 Apr 23;9:30. doi: 10.1186/s13229-018-0215-7 (PMC5914043; doi:10.1186/s13229-018-0215-7)
Supplement: Supplementary file 1 — Table S1. Levels of maternal blood mercury above the 80th centile found with the proxies for mercury exposure as used in Table 4. (DOCX 16 kb) [file 13229_2018_215_MOESM1_ESM.docx]

**Additional File 1**

**Prenatal mercury exposure and features of autism: a prospective study**

by

Jean Golding, Dheeraj Rai, Steven Gregory, Genette Ellis, Alan Emond, Yasmin Iles-Caven, Joseph Hibbeln, and Caroline Taylor

Table S1. Levels of maternal blood mercury above the 80^th^ centile found with the proxies for mercury exposure as used in Table 4.

**Supplementary Table 1**. Levels of maternal blood mercury above the 80^th^ centile found with the proxies for mercury exposure as used in Table 4.

| **Proxy for mercury exposure·** | **% (n) with Hg > 2·74 µg/L** | **% (n) with Hg < 2·74 µg/L** | **P value** | **Mean (SD) Hg** | **P value** |
| --- | --- | --- | --- | --- | --- |
| White Fish Frequency^a^ | | |  |  |  |
| Not at all | 10·2 (66) | 89·8 (578) |  | 1·63 (1·02) |  |
| Once in two weeks | 19·1 (266) | 80·9 (1130) |  | 2·09 (0·99) |  |
| >Once a week | 27·1 (385) | 72·9 (1038) |  | 2·35 (1·14) |  |
| P (2df) |  |  | *<0·0001* |  | *<0·0001* |
|  |  |  |  |  |  |
| Oily Fish Frequency^a^ | | |  |  |  |
| Not at all | 10·7 (158) | 89·3 (1321) |  | 1·75 (0·94) |  |
| Once in two weeks | 24·4 (278) | 75·6 (861) |  | 2·28 (1·08) |  |
| >Once a week | 33·3 (281) | 66·7 (564) |  | 2·50 (1·19) |  |
| P (2df) |  |  | *<0·0001* |  | *<0·0001* |
|  |  |  |  |  |  |
| Shell Fish Frequency^a^ | | |  |  |  |
| Not at all | 17·6 (487) | 82·4 (2279) |  | 2·02 (1·05) |  |
| Any | 33·0 (230) | 67·0 (467) |  | 2·49 (1·19) |  |
| P (2df) |  |  | *<0·0001* |  | *<0·0001* |
|  |  |  |  |  |  |
| Had amalgam fillings inserted in pregnancy | | |  |  |  |
| Yes | 26·7 (167) | 73·3 (458) |  | 2·34 (1·18) |  |
| No | 20·6 (448) | 79·4 (1723) |  | 2·11 (1·06) |  |
| P (1df) |  |  | *0·001* |  | *<0·0001* |
|  |  |  |  |  |  |
| Had amalgam fillings removed in pregnancy | | |  |  |  |
| Yes | 27·1 (112) | 72·9 (301) |  | 2·35 (1·02) |  |
| No | 21·1 (503) | 78·9 (1880) |  | 2·13 (1·11) |  |
| P (1df) |  |  | *0·007* |  | *0·0002* |
|  |  |  |  |  |  |
| Number of amalgams in mouth in pregnancy | | |  |  |  |
| 0 | 13·0 (27) | 87·0 (180) |  | 1·83 (1·16) |  |
| 1-3 | 14·5 (85) | 85·5 (501) |  | 1·93 (1·16) |  |
| 4+ | 25·6 (461) | 74·4 (1337) |  | 2·28 (1·05) |  |
| P (2df) |  |  | *<0·0001* |  | *<0·0001* |

^a^Amount consumed by mother as reported at 32 weeks gestation. All associations with P< 0·10 in bold
